# Comparison of nutritional properties of Stinging nettle (*Urtica dioica*) flour with wheat and barley flours

**DOI:** 10.1002/fsn3.259

**Published:** 2015-08-07

**Authors:** Bhaskar Mani Adhikari, Alina Bajracharya, Ashok K. Shrestha

**Affiliations:** ^1^ Department of Food TechnologyNational College of Food Science and TechnologyKathmanduNepal; ^2^ Nutrition and Food Science, School of Science and HealthHawkesbury Campus, University of Western SydneyPenrithNSW2751Australia

**Keywords:** Antioxidant activity, barley flour, nettle powder, polyphenol

## Abstract

Stinging nettle (*Urtica dioica. L*) is a wild, unique herbaceous perennial flowering plant with Stinging hairs. It has a long history of use as a food sources as a soup or curries, and also used as a fiber as well as a medicinal herb. The current aim was to analyze the composition and bioactive compounds in Nepalese Stinging nettle. Chemical analysis showed the relatively higher level of crude protein (33.8%), crude fiber (9.1%), crude fat (3.6%), total ash (16.2%), carbohydrate (37.4%), and relatively lower energy value (307 kcal/100 g) as compared to wheat and barley flours. Analysis of nettle powder showed significantly higher level of bioactive compounds: phenolic compounds as 129 mg Gallic acid equivalent/g; carotenoid level 3497 *μ*g/g; tannin 0.93 mg/100 g; anti‐oxidant activity 66.3 DPPH inhibition (%), as compared to wheat and barley. This study further established that nettle plants as very good source of energy, proteins, high fiber, and a range of health benefitting bioactive compounds.

## Introduction

Stinging nettle (*Urtica dioica, L. Urticaceae*) is a ubiquitous herb which is available in large part of the world. *Urtica dioica* is a moderately shade‐tolerant species, which occurs on most moist or damp, weakly acid or weakly basic, richly fertile soils. Its stems and leaves are densely covered with Stinging hairs, which release potentially pain‐inducing toxins, is rarely eaten by castles and rabbits (Taylor, 2009). This species is known as tenacious weeds, able to live in the toughest conditions, and notoriously known for inflicting pain. From ancient times, the fresh Stinging nettle is used for flailing arthritic or paralytic limbs with fresh Stinging nettle to stimulate circulation and bring warmth to joints and extremities in a treatment known as “urtication” (Green, 1820). Ancient Egyptians also reportedly used the infusion for the relief of arthritis and lumbago (Harrison 1966). Above mentioned practice of urtication or rubefaction became a standard in folk medicine as a remedy for arthritis, rheumatism, and muscular paralysis and is perhaps the most ancient medicinal use of Stinging nettle (Upton 2013). Nettle can be used to foster health and vitality of the people. Due to the nutritional and functional qualities of nettle, it has been utilized to alleviate symptoms associated with allergic rhinitis and improve oxidative stability in brine anchovies. It is also rich in fatty acids, carotenoid, and phenolic compounds, while its extracts have been reported to improve oxidative stability in brined vegetables (Rutto et al. 2013).

A comprehensive proximate analysis showed the shoots harvested from Stinging nettle (Shoot) showed close to 90% moisture and rests are proteins (3.7%), fat (0.6%), ash (2.1%), dietary fiber (6.4%), total carbohydrate (7.1%), and total calories (45.7 kcal/100 g) (wet basis). Besides, Stinging nettle (shoot) contains vitamin A, vitamin C, calcium, iron, sodium, and rich fatty acid profile (Rutto et al. 2013). Farag et al. (2013) studied the geographic, taxonomical and morphological diversity, genetics, etc., under control conditions. *Urtica dioica* is the only species of Urtica to be cultivated commercially for pharmaceutical purposes, and the commercial extraction of chlorophyll and stem fibers. He further reported *U. dioica* as a good source of flavonoids, phenylpropanoids, and caffeic acid analogues. Besides, the use of Nettle extract for rheumatism, eczema, allergic rhinitis, and arthritis is well studied (Harrison 1966; Upton 2013).

Stinging nettle is rarely domesticated due to its sting but the species remains popular as food and medicines in poor countries like Nepal (Uprety et al. 2012). From the centuries, in the foothills of Nepal's Himalayas, the Himalayan Stinging nettle has naturally grown in the wild. Recently, founder of Himalayan Wild Fibers, is in the process of developing the nettle fiber industry with the local community. It is expected to help in the development of strong fiber that would create work and income to many Nepalese and bring a durable and sustainable textile to the market (Tree hugger).

In Georgia, a meal of boiled Stinging nettle seasoned with walnut is common. Romanians use sour soup made from fermented wheat bran vegetables and green nettle leaves harvested from young plants (Costa et al. 2013). However, one of the most underutilized and neglected crops are now getting attention on their commercial utilization due to its nutrition and functional properties. Production and processing of different products from it will also support to uplift the economic status of the local people from third world countries (Palikhe 2012). Stinging nettle is very popular as a vegetable in a range of countries, particularly among the lower socioeconomic people. More work is needed to learn the nutritive value of Nepalese cultivars. Stinging nettle is consumed primarily as a boiled or cooked fresh vegetable whereby it is added to soups, cooked as a pot herb, or used as a vegetable complement in dishes. Although popular in Nepal, there is almost no study on Stinging nettle (Palikhe 2012).

In Nepal, wheat and barley are two most consumed cereal grains, after rice. These are the major source of starch, fiber, proteins, lipid, minerals, etc. Barley grain is reported to be effective in lowering blood cholesterol because of its high *β*‐glucan content, 2–9% (Hassan et al. 2012). The recent approval of soluble barley beta‐glucan health claims by the Food and Drug Administration of the USA for lowering blood cholesterol level could further boost food product development from barley and consumer interest in eating these food products (Yamlahi and Ouhssine 2013). Wheat is the staple diet for a majority of global population, containing carbohydrates, protein, minerals, B‐group vitamins, and dietary fiber, etc. Starch is the major component of wheat, providing calorie as well as the inner bran coats, phosphates, and other mineral salts; the outer bran, the much‐needed roughage the indigestible portion that helps easy movement of bowels; the germ, vitamins B and E. Kumar et al. (2011) have comprehensively reviewed the nutritional content and medicinal properties of wheat.

Nepal is one of the developing countries in the world but it is rich in flora and fauna. Stinging nettle is one of the most popular wild edible plants (WEP) that provide staple and supplement foods. Often these WEPs become the top cash income to the local communities which contribute to food security to the region. However, there is hardly any work on the composition and nutritional properties of Stinging nettle in Nepal. Therefore, this work studied the nutritional and functional properties of stinging nettle dried powder. Besides, this work also compared the properties of Stinging nettle powder with wheat as well as barley flour.

## Materials and Methods

### Raw material collection and preparation

Stinging nettle (*Urtica dioica)* leaves were collected from Kirtipur, Nepal. The collected samples were carefully carried afresh to the laboratory for chemical analysis. The Stinging nettle leaves were cleaned (washed) so that the foreign particles are removed. The leaves were blanched for 1 min at 80°C in wet condition. The leaves were then drained, placed on the trays and so as to remove excess water. Then the trays were put inside the cabinet drier for drying at 60°C for 2 days, till the crispy texture was observed. Dried leaves were ground in a coffee grinder and sieved through the 80 size mesh making into a fine Nettle powder as done previously (Palikhe 2012). Barley and wheat flours were purchased from the local market. All dried flours were analyzed for moisture content and transferred into the hermitically sealed container.

### Chemical analysis

All three flour samples *viz*., wheat, barley and Stinging nettle were immediately analyzed in triplicates. Moisture content was measured by oven drying at 100°C until the constant weight was reached. Total crude oil of all three samples was extracted using hexane in Soxhlet System HT2 Texator, (Sweden). Total ash values were obtained by incineration of flour samples for overnight at 550°C at minimum 6 h (AOAC, 2005). Calcium content was measured by precipitation as calcium oxalate, dissolving in concentrated sulfuric acid and titration with standard potassium permanganate (Ranganna 2001). Iron was determined by converting iron present in foods into ferric form and treating thereafter with potassium thiocyanate to form the red ferric thiocyanate which is measured by colorimetry at 480 nm (Ranganna 2001). Total carbohydrate content was measured by the difference method. All chemical analyses were conducted by the methods as recommended by Ranganna (2001).

A number of functional properties of above samples were also analyzed. The antioxidant activity (AA, DPPH inhibition %) was determined by the method described by Nuengchamnong et al. (2009). Total polyphenol (TP, mg GAE/g) and carotenoids were determined as per the method described by Ranganna (2001). Tannin as an anti‐nutritional factor was determined according to AOAC (2005).

### Data analysis

Data were statistically processed by Gen Stat for analysis of variance (ANOVA), Microsoft Excel‐2007 for analysis. Means of data were separated whether they are significant or not by using LSD (least square difference) method at 5% level of significance.

## Results and Discussion

### Proximate analysis of raw materials

Wheat and barley flours were purchased from the local market but nettle was processed into fine powders as mentioned in the methodology section. The chemical analyses of wheat flour, barley flour and nettle powder were carried out and results are presented in dry basis. The mean values of chemical composition of wheat flour, barley flour, and nettle powder are presented in Table 1. The initial moisture content of the leaves was not measured. However, previous studies have shown that the Nettle plant contains relatively high level of moisture of, for example, 89% (Rutto et al. 2013) and 84.4% (Mishra 2007). The moisture content of wheat flour was 12.4% which is common in commercial wheat flour as previously reported by Kent and Evers (2004). Barley flour had similar moisture content, 12.2%. After cabinet drying of leaves followed by grinding, the moisture content of the Nettle powder was reduced significantly to 7.0% (Table 1).

**Table 1:** Chemical composition of wheat, barley, and nettle powders^a^

Parameters	Wheat flour	Barley flour	Nettle powder
Moisture (%)	12.37 ± 0.25	12.2 ± 0.19	7.04 ± 0.77
Crude protein (%,db)	10.6 ± 0.23	11.84 ± 0.09	33.77 ± 0.35
Crude fiber (%,db)	0.65 ± 0.13	1.03 ± 0.08	9.08 ± 0.14
Crude fat (%,db)	1.68 ± 0.23	1.73 ± 0.67	3.55 ± 0.06
Total ash (%,db)	0.56 ± 0.07	3.6 ± 0.08	16.21 ± 0.54
Carbohydrate (%,db)	86.51 ± 0.27	81.8 ± 0.08	37.39 ± 0.72
Calcium (mg/100 g)	18.94 ± 0.08	17.51 ± 0.26	168.77 ± 1.47
Iron (mg/100 g)	3.37 ± 0.29	3.63 ± 0.11	227.89 ± 0.21

db, dry basis.

a Values are mean ± Standard deviation of triplicates.

Protein content of the ground wheat, barley and Stinging nettle were 10.6%, 11.8%, and 33.8% (db), respectively. Analytical data of the nettle powder exhibits about 3 times protein level as compared to the traditional source of cereal proteins, *that is,* rice, wheat, and barley. Previous study also showed a relatively higher amount of protein content, 33.6% (dry basis) in the Nettle powder. Considering a higher level of protein in Nettle powder, this species expected to supply higher concentrations of essential amino acids. Besides, it has a better amino acid profile than most of the other leafy vegetables (Rutto et al. 2013).

Rutto et al. (2013) has reported relatively higher amounts of all essential amino acid content in Stinging nettle, except leucine and lysine. Nettle leaf flour has been incorporated in many recipes, for example, bread, pasta, and noodles dough that suggest it could be used as a protein‐rich supplement in starchy diets associated with poor and undernourished population like Nepal. As compared to the conventional source of proteins, Nettle powder contains 3.2 and 2.9 times greater amount of proteins as compared to wheat and barley flours, respectively. Nettle powder has one of the richest sources of crude fiber (9.1%, db) (Table 1). The amount of crude fiber in the nettle powder is significantly higher than most of the cereals and other plant foods, more than 9 times higher as compared to wheat and barley flour. Published literatures showed the Nettle powder has 6.4% (db) crude fiber (Rutto et al. 2013). The level of crude fat is relatively low at 3.6% (db), but this value is still higher than wheat (1.7%) and barley flour (1.7%).

Stinging Nettle is rich in minerals. Current study showed the Nettle powder contained 16.2% (db) ash content which is much higher than conventional cereals (Table 1). Rutto et al. (2013) reported that the total ash content of Nettle powder is 19.1% (db) or 2.1% in wet basis. The higher level of minerals in Nettle is also demonstrated by higher level of calcium (169 mg/100 g) and iron (277 mg/100 g) (Table 2). Once again, these values are much higher than those from wheat and barley flours. USDA data showed the Nettle powder contains 4% calcium (db), 2.8% (db) potassium followed by phosphorus, magnesium and traces of iron, sodium and zinc (USDA, 2008). Based on this data, Nettle powder probably is one of the richest sources of minerals among the plant foods. In comparison, wheat flour and barley flour have much lower total ash content, 0.6% and 3.6%, respectively.

**Table 2:** Analysis of wheat flour, barley flour, and nettle powder^a^

Parameters	Wheat flour	Barley flour	Nettle powder
Tannin content (% as is)	ND	0.53 ± 0.03	0.93 ± 0.01
Total polyphenol (mg GAE/g, db)	1.31 ± 0.01	1.76 ± 0.01	128.75 ± 0.21
Antioxidant activity (DPPH inhibition, % as is)	23.72 ± 0.45	28.64 ± 0.03	66.3 ± 0.12
Carotenoids (*μ*g/g, db)	320.05 ± 0.08	382.3 ± 0.56	3496.67 ± 0.56
Calorific value (kcal/100 g)	381.93 ± 0.05	369.68 ± 0.84	307.24 ± 0.13

db, dry basis; ND, not detected.

a Values are mean ± standard deviation of triplicates.

Nettle powder contained the lowest amount of carbohydrate (37.4%, db) as compared to regular cereals, for example, wheat (86.5%) and barley (81.8%). It shows the Nettle powder is much less glycemic as compared to the conventional sources of plant foods such as cereals and tuber in particular. Table 1 shows the crude fiber (9.1% db) forms a significant component of Nettle powder. Rutto et al. (2013) shows the carbohydrate content of Nettle powder is close to the currently analyzed value, 7.1% db. The calorific value of wheat flour was higher, that is, 381.9 kcal/100 g than barley flour and nettle powder which were 369.7 kcal/100 g and 307.2 kcal/100 g, respectively. This also shows the Nettle powder is low in calorie as compared to conventional cereals.

Carbohydrate levels in the nettle powder decreases with increase in the protein content, fiber, ash, and fat as shown in Table 1. The results agree to the report given by Palikhe (2012) Thapaliya (2010). Therefore, the current finding showed that the use of nettle powder and barley in bakery products likely to increase the protein, ash and fiber whereas decrease in calorific value as well as increase in bioactive compound (discussed later). The incorporation of barley flour in the cereal based food products such as biscuits, breakfast cereals, noodles, etc., potentially lower the blood cholesterol, cardiovascular and other diet related because of soluble *β*‐glucan (Hassan et al. 2012).

### Functional properties of raw materials

Nettle plant and its associated products are reported to be rich in a number of bioactive compounds (Knipping et al. 2012; Johnson et al. 2013; Rutto et al. 2013). It has been reported that the natural phenolic compounds play an important role in cancer prevention and treatment. Phenolic compounds from medicinal herbs and dietary plants include phenolic acids, flavonoids, tannins, stilbenes, curcuminoids, coumarins, lignans, quinones, and others. Various bioactivities of phenolic compounds are responsible for their chemopreventive properties (e.g., antioxidant, anticarcinogenic, or antimutagenic and anti‐inflammatory effects) and also contribute to their inducing apoptosis by arresting cell cycle, regulating carcinogen metabolism and ontogenesis expression, inhibiting DNA binding and cell adhesion, migration, proliferation or differentiation, and blocking signaling pathways (Knipping et al. 2012; Johnson et al. 2013).

Table 2 shows the nettle powder contained relatively higher level of bioactive compounds, for example, tannin, total polyphenol (TP), antioxidant activity (AA), carotenoid, and total caloric value as compared to wheat and barley flours. The total phenolic content of nettle powder was 129 mg GAE (Gaelic Acid Equivalent)/g, which is much higher than the wheat flour (1.3 GAE/g) and barley flour (1.7 GAE/g). One of the quantitative analysis of plant phenolics in Nettle plant showed only 21 of the 45 compounds in levels above the reliable quantification limit (Orcic et al. 2014). Natural phenolic compounds reported to play important role in cancer prevention and treatment. A comprehensive review showed the compounds from medicinal herbs such as Nettle plant and dietary plants include phenolic acids, flavonoids, tannins, curcuminoids, coumarins, lignans, etc. (Huang et al. 2010).

The carotenoid content of wheat flour, barley flour and nettle powder were 320, 382.3, and 3496.7 *μ*g/g, respectively. Nettle powder appeared to have almost ten times higher amount of carotenoid as compared to wheat flour and barley flour (Table 2). According to Rutto et al. (2013), the blanched nettle at 98°C for 1 min contained 4689 *μ*g/g amount of carotenoids. It seems that in both cases, the most significant reductions might have occurred due to the longer exposure to heat during the drying process. It has been reported that the amount of vitamin A, iron, and calcium are significantly affected by the heat (Rutto et al. 2013).

Carotenoids are the precursors of vitamin A and similar compounds. *β*‐carotene is one of most commonly known carotenoids which is a potent antioxidant as well as a dietary factor for growth. It is a precursor of vitamin A that has important role in vision, as the prosthetic group of the light sensitive proteins in retina, and a major role in the regulation of gene expression and tissue differentiation (Bender, 2003). Deficiency of vitamin A is a major public health problem around the world. The prevention of vitamin A deficiency is one of the three micronutrient priorities of the World Health Organization (WHO), others are iron and iodine.

Tannins (Polyphenols) occur in cereals, especially in the seed coat (Reilly et al. 2009). The tannin content of barley flour and nettle powder was 0.53 and 0.93 mg/100 g, respectively, whereas, no tannin was observed in the wheat flour. Polyphenols occur in cereals and these form complexes with proteins and inhibition of digestive enzymes. Nettle powder had higher level of anti‐oxidant activity of 66.3 DPPH inhibition (%) as compared to wheat flour 23.72 DPPH inhibition (%) and barley flour (28.64 DPPH inhibition (%). Higher level of antioxidant activity (AA) was also observed in Nepalese nettle powder. Current data showed similar amino acid value in Nepalese nettle, as reported by Thapaliya (2010).

A prospective, randomized, double‐blind, placebo‐controlled, crossover study showed the *Urtica dioica* reported to have beneficial effects in the treatment of symptomatic benign prostatic hyperplasia (BPH). Further clinical trials should be conducted to confirm these results before concluding that *Urtica dioica* is effective.

## Conclusions

Stinging nettle (*Urtica dioica*) is a common herb and its stem and leaves are densely covered with Stinging hairs that inflict pain. It is eaten as a curry, sour soup, vegetable complement in dish, etc. Stinging nettle has a great medicinal value such as relieve of arthritis, rheumatism, muscular pain, etc. Nettle powder contains high amount of protein (38%), crude fiber (9%), total ash (16.2%), calcium (0.17%), iron (0.23%), and relatively low in carbohydrate (37%). As compared to barley and wheat flour, it has much higher protein, crude fiber, fat, ash, calcium and iron, and low in glycemic index. Besides, it has excellent health enhancing functional properties as compared to conventional grains. As compared to barley and wheat, it has much higher level of tannin content, total polyphenol, antioxidant activity, carotenoids, and lower calorific value. Bioactivities of these functional components may play important role in arthritis, rheumatism, muscular paralysis, potentially cancer prevention, etc.

## Conflict of Interest

None declared.
